# Oyster crude polysaccharides attenuates lipopolysaccharide-induced cytokines production and PPARγ expression in weanling piglets

**DOI:** 10.1186/s40064-016-2319-x

**Published:** 2016-05-23

**Authors:** Guangwen Yin, Juhui Huang, Maotao Ma, Xun Suo, Zhijian Huang

**Affiliations:** Engineering Laboratory of Animal Pharmaceuticals and College of Animal Science, Fujian Agriculture and Forestry University, Fuzhou, 350002 Fujian Province China; National Animal Protozoa Laboratory and College of Veterinary Medicine, China Agricultural University, Beijing, 100193 China

**Keywords:** Oyster polysaccharides, Immune stress, Cytokines, PPARγ, Piglets

## Abstract

This study evaluated whether oyster crude polysaccharides (OPS) attenuates lipopolysaccharide (LPS)-induced immune stress in weanling piglets. Thirty healthy crossbred piglets (28 ± 1 days old) were randomly divided into five groups (6 piglets/group). Blank control and LPS groups were fed with the basal diet, while low, medium and high dose of OPS groups were fed with the basal diet supplemented with 0.5, 0.8 and 1.2 % OPS, respectively, for 30 days. LPS group, as well as low, medium and high dose of OPS groups were then injected intraperitoneally with LPS (100 μg/kg body weight), whereas the blank control group was given phosphate buffered saline. The concentrations of TNF-α, IL-1β and IL-6 in plasma were detected by ELISA. The mRNA levels of PPARγ in liver, spleen, adrenal gland and thymus were evaluated by quantitative real-time PCR. The results showed that compared with the blank control, LPS treatment significantly increased plasma IL-1β, IL-6 and TNF-α levels, which was significantly attenuated by supplementing 0.5, 0.8 or 1.2 % OPS in the diet. In addition, LPS significantly induced expression of PPARγ mRNA in liver, spleen, adrenal gland, and thymus, which was blocked by adding OPS regardless of the doses. These results indicate that dietary supplementation of OPS was able to alleviate the immune stress induced by LPS.

## Background

Proinflammatory cytokines including interleukin-1 (IL-1), interleukin-6 (IL-6), and tumor necrosis factor-α (TNF-α) promote systemic inflammation (Cagiola et al. 2006; Cook et al. 2011; Johnson 1997; Webel et al. 1997). Because of their proinflammatory activity, these cytokines may make a disease deteriorated by producing inflammation and tissue damage, leading to shock or death (Baggio et al. 2005; Netea et al. 2003). Previous studies have shown that in response to LPS challenge, the proinflammatory cytokines can be induced, reducing feed intake and growth in pigs (Johnson and von Borell 1994). Lipopolysaccharide (LPS), an endotoxin, is the major component of the outer membrane of Gram-negative bacteria such as *Escherichia coli* (*E. coli*). Administration of LPS in animals is a well-documented model for immune stress (Escribano et al. 2014). LPS exerts its effect through stimulating numerous cell types, such as macrophages, to produce pro-inflammatory cytokines, including TNFα, IL1β and IL6 (Webel et al. 1998; Dinarello 1996).

Peroxisome proliferator activated receptor gamma (PPARγ), a transcriptional factor, belongs to the ligand-activated nuclear receptor super family, and is expressed virtually in all kinds of cells, including macrophages, dendritic cells, T and B cells (Yang et al. 2008). PPARγ was originally found to regulate adipocyte differentiation and lipid metabolism (Liu et al. 2008; Monsalve et al. 2013; Mueller et al. 2003). However, accumulating evidence has implicated that PPARγ also plays an important role in the regulation of proinflammatory cytokine production, and contributes to the therapeutic effects on inflammatory diseases (Jacob et al. 2007; Kim et al. 2012). Activation of PPARγ has been shown to inhibit cytokine production by preventing activation and translocation of NF-κB (Li and Palinski 2006; Touyz and Schiffrin 2006).

The pacific oyster *Crassostrea gigas* (*C. gigas*) is a common aquaculture species widely consumed in many countries. The oyster is a rich source of dietary protein and other nutrients, such as carbohydrates, lipids, polyunsaturated fatty acids and minerals (Pennarun et al. 2003; Cheng et al. 2013). It has been described that the extract of *C. gigas* exhibits antioxidant activity (Watanabe et al. 2012). Recently, a phenolic antioxidant from the oyster has been identified (Watanabe et al. 2012; Yoshikawa et al. 1997). Strong evidence has demonstrated that polysaccharides derived from yeast and medicinal mushrooms possess immunomodulatory activity (Cheng et al. 2013). Feeding β-glucans can stimulate both specific and nonspecific immune responses in mice (Yun et al. 2003). Additionally, polysaccharides from aloe can also inhibit the inflammatory response (Strickland et al. 1999).

Although many studies have shown the immunomodulatory activities of polysaccharides, most of them focus on fungal polysaccharides. To date, limited evidence is available pertaining to the immunomodulatory activity of polysaccharides derived from animal origins. As the extract of *C. gigas* contains a considerable amount of carbohydrates (Cheng et al. 2013), we hypothesized that OPS may alter the immune response in weanling piglets. To test this hypothesis, here we investigated whether OPS attenuates LPS-induced cytokines production and PPARγ expression in weanling piglets.

## Methods

### Animals and treatments

Oyster crude polysaccharide (OPS) was provided by Addison Biological Technology (Beijing, China). Crossbred healthy piglets, weighing 9.91 ± 1.38 kg and aged 28 ± 1 days, were castrated and randomly divided into five groups (namely blank control, LPS control, low OPS, medium OPS, high OPS groups) with six replicates per group. The piglets in the blank control group and LPS group were only fed the basal diet (Table 1), whereas the animals in the OPS low, medium and high groups were fed the basal diet supplemented with 0.5, 0.8 and 1.2 % OPS, respectively. All groups were fed with the above diets for 30 days. Then the animals were weighed and intraperitoneally injected once with either phosphate buffered saline (PBS) (only for blank control group) or LPS (100 μg/kg body weight). The LPS (*E. coli* serotype O55:B5 phenol extract; Cat.# L-2630, Sigma-Aldrich, St. Louis, MO) was dissolved in PBS solution (Cat.# P-4417, Sigma-Aldrich) to make 0.4 mg/ml LPS solution. Three hours post injection, all piglets were euthanized for blood and tissue sampling. Six piglets from each treatment were sacrificed after feed deprivation for 12 h by injecting 4 % sodium pentobarbital solution (40 mg/kg body weight) for the collection of tissue samples on day 30 post weaning. The animal protocol was approved by the Animal Care Committee of Fujian Agriculture and Forestry University.

**Table 1:** Composition of experimental diets

Item		Item	
Ingredient, %		Nutrient level, %	
Corn	49.8	Crude protein	19.52
Farina	9	Crude fiber	2.82
Liquid feed oil	2	Crude fat	4.72
Soybean meal	21.3	Calcium	0.75
Fermented soybean meal	5	Phosphorus	0.54
Fish meal	3	Dry matter	87.53
Glucose	2	Digestive energy (MJ/kg)	14.3
Sugar	2	Lysine	1.33
Vitamin-mineral mix^a^	6		

^a^The vitamin and mineral premix provided the following (per kg of diet): vitamin A, 10,165 IU; vitamin D3, 1007 IU; vitamin E, 75 IU; Zn, 2133 mg; Fe, 296 mg; Mn, 45 mg; Cu, 134 mg; Co, 0.31 mg; and Se, 0.5 mg

### Blood sampling and preparation

Blood samples (10 ml per piglet) were collected from the precaval vein. Each sample was drawn into a plastic tube with liquaemin and centrifuged at 3000×*g* for 10 min at room temperature. The plasma obtained was stored at −20 °C until analysis for cytokines.

### Tissue sampling and preparation

Approximately 1 g of liver, adrenal gland, thymus or spleen was collected immediately, frozen in liquid nitrogen, and stored at −80 °C until the extraction of total RNA.

### Analysis of IL-1β, IL-6, and TNF-α concentrations

Concentrations of IL-1β, IL-6, and TNF-α in the plasma were measured using ELISA kits (Cusabio Biotech Co., Wuhan, China), according to the manufacturer’s instructions. The sensitivity of all assays was 3 pg/ml.

### Quantitative real-time PCR

Total RNA was extracted from the samples using Trizol reagent (Invitrogen, Carlsbad, CA). The final RNA was eluted in an appropriate amount of 0.1 % diethyl pyrocarbonate (DEPC) treated water (Sigma-Aldrich). For each sample, the integrity of RNA extracted was confirmed in agarose gel electrophoresis by staining with ethidium bromide and visualizing under UV light. The amount of RNA extracted was determined, and its purity (OD260/OD280 ratio between 1.8 and 2.2) was verified using an ND-1000 spectrophotometer (NanoDropTechnologies, Wilmington, DE, USA). The cDNA was synthesized using random primers and a High Capacity cDNA Reverse Transcription Kit (Applied Biosystems, Foster City, CA, USA). The primer pairs used for analysis of specific genes (Table 2) were designed with the PerlPrimer software (perlprimer.sourceforge.net). Quantitative real-time PCR was performed on the 7500 Real Time PCR System (Applied Biosystems) with a program of 50 °C for 2 min, 95 °C for 10 min and 40 cycles of 95 °C for 15 s, and 60 °C for 1 min. For each sample, template copy numbers were internally normalized with their respective input control. Relative expression was calculated as the ratio of template copy numbers of a sample relative to the naive control after normalizing to their respective isotype control β-actin (Huang et al. 2011).

**Table 2:** Primers for real-time PCR

Gene	Primers	Product Size (bp)	Efficiency (%)
*PPARγ*	F: CTGACCAAAGCAAAGGCG	191	102
	R: TCCACGGAGCGAAACTGA		
*β*-*actin*	F: CCAGGTCATCACCATCGGC	152	103
	R: TGGCGTAGAGGTCCTTGCG		

### Statistical analysis

Values were expressed as mean ± SD. Data were subjected to one-way analysis of variance (ANOVA) to determine whether significant differences occurred in pig fed the different diets. If a significant difference was identified, differences among means were compared by Tukey’s multiple range tests (P < 0.05). Statistical analysis was performed using the Statistica software package (Version 6.0, Statsoft, Tulsa, OK, USA).

## Results

### OPS attenuates LPS-induced cytokines production in piglets

As shown in Table 3, treatment of piglets with LPS significantly elevated the plasma concentrations of IL-1β, IL-6 and TNF-α (*P* *<* 0.05), compared with the blank control (PBS), suggesting that LPS did induce immune stress. Of interest, the LPS-induced immune stress was significantly attenuated by adding low (0.5 %), medium (0.8 %), or high dose of OPS (1.2 %) (*P* < 0.05). There was no significant difference between medium OPS group and low OPS group, in terms of the levels of the proinflammatory cytokines. To our surprise, 0.5 and 0.8 % OPS inhibited LPS-induced IL-6 and TNF-α more potently than 1.2 % OPS (*P* < 0.05).

**Table 3:** Effect of OPS on LPS-induced protein expression of IL-1β, IL-6 and TNF-α in the plasma of piglets

Group	IL-1β	IL-6	TNF-α
Blank control	12.50 ± 2.80^b^	2.14 ± 0.06^c^	81.55 ± 5.50^c^
LPS control	32.62 ± 12.53^a^	2.48 ± 0.09^a^	166.65 ± 20.88^a^
Low OPS	11.68 ± 3.27^b^	2.09 ± 0.13^c^	77.62 ± 7.82^c^
Medium OPS	11.03 ± 2.06^b^	2.02 ± 0.07^c^	86.14 ± 3.83^c^
High OPS	12.14 ± 2.55^b^	2.34 ± 0.06^b^	107.85 ± 6.61^b^

Different letters in the same rank represent significant difference between the treatments (*P* < 0.05), while the same letter in the same rank means no significant difference between the treatments (*P* > 0.05). The units for IL-1β, IL-6 and TNF-α in the table (pg/ml)

### OPS attenuates LPS-induced expression of PPARγ mRNA in piglets

**Fig. 1:**
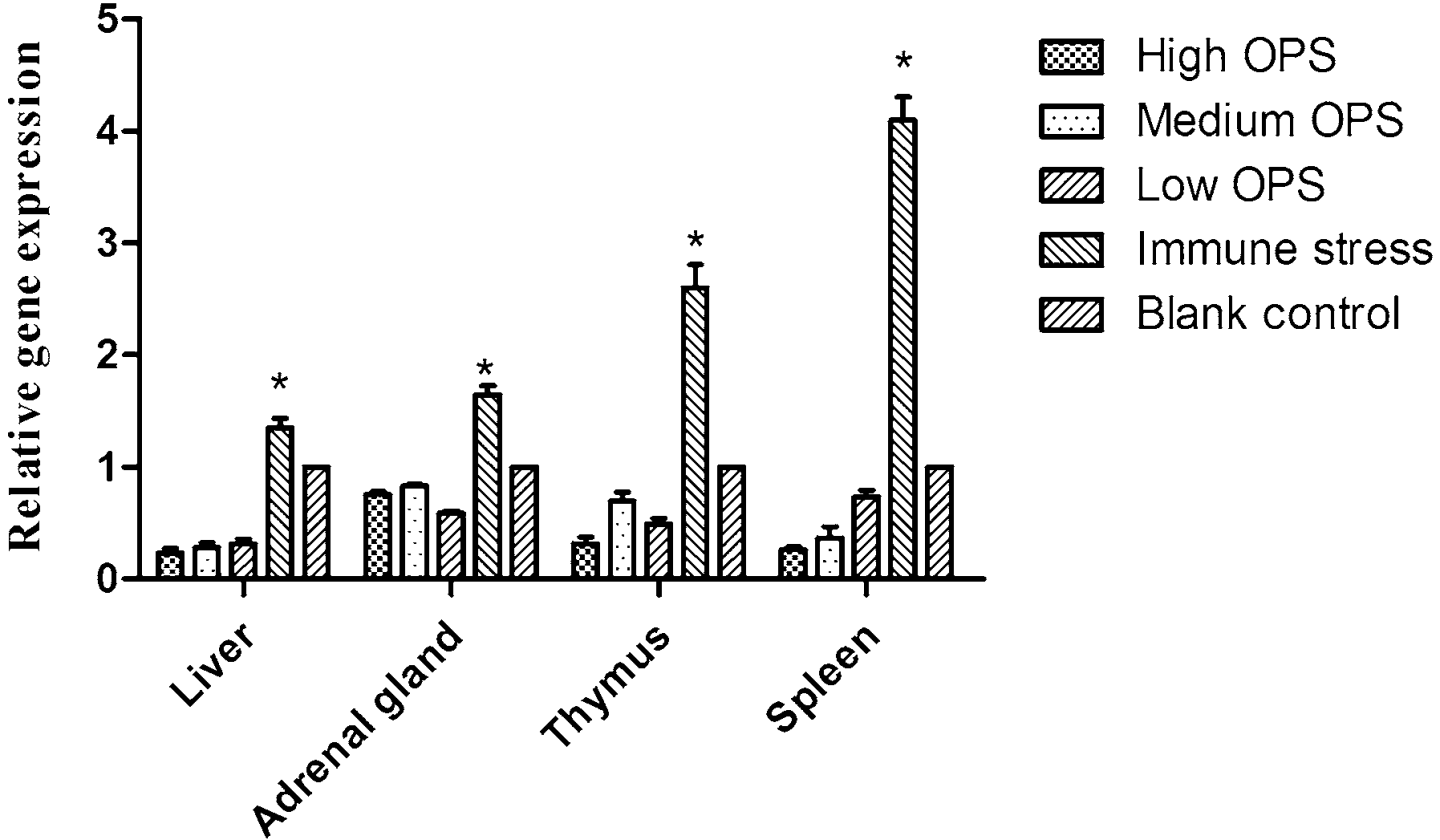
Effect of OPS on LPS-induced PPARγ mRNA expression in liver, adrenal gland, thymus, and spleen of piglets. Experiments were performed in duplicate and repeated in at least three independent experiments using tissues from 6 individual piglets. Results are expressed as mean ± SEM. *P < 0.05

As shown in Fig. 1, treatment of piglets with LPS significantly increased expression of PPARγ mRNA in liver, adrenal gland, thymus and spleen, compared with the blank control (PBS) (*P* < 0.05). LPS-induced PPARγ expression was significantly attenuated by adding low (0.5 %), medium (0.8 %), or high dose of OPS (1.2 %). It appeared that the effect of OPS on LPS-induced PPARγ expression was independent of concentrations of OPS used in the experiment.

## Discussion

Immune stress induces production of proinflammatory cytokines in the body, which can decrease growth rate, and accelerate degradation of body protein. Therefore, immune stress can cause significant economic loss to livestock producers (Tuchscherer et al. 2002). Previous studies have shown that the proinflammatory cytokines TNFα, IL-6, and IL-1β are elevated in plasma in response to peripheral LPS injection in weaned pigs (Chirullo et al. 2015; Webel et al. 1997). Here we found that an intraperitoneal injection of LPS in weaned piglets increased the levels of TNFα, IL-6, and IL-1β in the plasma, which is consistent with the previous findings (Wright et al. 2000; Webel et al. 1997). Interestingly, LPS-induced elevation of IL-1β, IL-6 and TNFα levels was significantly attenuated by supplementing OPS in the diet, indicating that OPS may play an anti-inflammatory role in the immune stress caused by LPS. OPS may reduce the expression of TLR4, a receptor for LPS, on immune-related cells, then attenuate the release of proinflammatory cytokines. In the present study, we noticed that the inhibitory effect of the high dose (1.2 %) of OPS on LPS-induced cytokines was weaker than that of the low (0.5 %) or the medium dose (0.8 %) of OPS. Our explanation is that high dose of OPS may change the palatability of the diet, and the feed intake of pigs, leading to the reduced absorptive capacity of OPS. Further research is needed to address this question.

Recently, the anti-inflammatory properties of the nuclear hormone receptor family known as peroxisome proliferator-activated receptors (PPARs) have emerged, although they were originally found to be implicated in obesity, diabetes, and atherosclerosis. PPARγ can be activated by specific agonists, and then translocate to the nucleus to regulate the transcription of specific genes. Activation of PPARγ can protect cells from injury by its anti-inflammatory and immunomodulatory effects (Belvisi and Hele 2008). Growing evidence suggests that the benefits of PPARγ agonists may also be derived from their anti-inflammatory properties (Celinski et al. 2013; Hanks et al. 2010). To date, however, limited evidence is available pertaining to the anti-inflammatory properties of PPARγ in pigs. We found that LPS was able to induce the expression of PPARγ in many tissues of piglets. The high level of expression of PPARγ mRNA induced by LPS implies that PPARγ may regulate inflammation in pigs. The trend was in line with the concentrations of the proinflammatory cytokines in the plasma. Our result is consistent with the previous report that LPS can increase the expression of PPARγ in the white blood cells of pigs (Leininger et al. 1999). In the present study, we found that pigs fed OPS had lower expression of PPARγ, indicating that OPS may play an important role in negatively regulating the expression of PPARγ and the production of the pro-inflammatory cytokines. OPS may influence the expression of PPARγ and TLR4 on monocytes or macrophages, then regulate the production of proinflammatory cytokines after LPS-stimulation. Further research is needed to shed the light on the mechanisms whereby OPS reduces th expression of proinflammatory cytokines.

## Conclusion

In conclusion, dietary supplementation of OPS was able to alleviate the immune stress induced by LPS, and the observed effects after the administration of OPS are concentrations used in the experiment independent.
